# Modulation of the cough reflex by GABA_A_ receptors in the caudal ventral respiratory group of the rabbit

**DOI:** 10.3389/fphys.2012.00403

**Published:** 2012-10-18

**Authors:** Elenia Cinelli, Fulvia Bongianni, Tito Pantaleo, Donatella Mutolo

**Affiliations:** Dipartimento di Scienze Fisiologiche, Università degli Studi di FirenzeFirenze, Italy

**Keywords:** GABA_A_ receptors, caudal ventral respiratory group, cough reflex, sneeze reflex, expiratory activity, expiratory neurons, control of breathing

## Abstract

We have previously shown that the caudal ventral respiratory group (cVRG) is a possible site of action of some antitussive drugs and plays a crucial role in determining both the expiratory and inspiratory components of the cough motor pattern. In addition, it has been reported that medullary expiratory neurons of the cVRG are subject to potent GABAergic gain modulation. This study was devoted to investigate the role of cVRG GABA_A_ receptors in the control of baseline respiratory activity and cough responses to mechanical and chemical (citric acid) stimulation of the tracheobronchial tree. To this purpose, bilateral microinjections (30–50 nl) of bicuculline or muscimol were performed into the cVRG of pentobarbital sodium-anesthetized, spontaneously breathing rabbits. Bicuculline (1 mM) increased peak abdominal activity and respiratory frequency due to decreases in T_E_. Cough responses were potentiated mainly owing to increases in the cough number. The recovery was observed within ~2 h. On the contrary, muscimol (0.3 mM) abolished abdominal activity and decreased respiratory frequency due to increases in T_E_. In addition, cough responses were progressively reduced and completely suppressed within ~20 min. Partial recovery of cough responses was achieved after ~3 h or within ~5 min following bicuculline microinjections at the same locations. The sneeze reflex induced by mechanical stimulation of the nasal mucosa persisted following bicuculline and muscimol microinjections. However, the number and intensity of expiratory thrusts were enhanced by bicuculline and suppressed by muscimol. The results provide evidence that a potent GABA_A_-mediated inhibitory modulation is exerted at the level of the cVRG not only on respiratory activity, but also on cough and sneeze reflex responses.

## Introduction

Cough is one of the most important airway defensive reflexes (Korpáš and Tomori, 1979). It is well known that tracheobronchial rapidly adapting receptors (RARs) are involved in its mediation, while the role of bronchopulmonary C-fibers and Aδ-nociceptive pulmonary afferent fibers is controversial (Coleridge and Coleridge, 1986; Widdicombe, 1998; Lee and Pisarri, 2001; Sant'Ambrogio and Widdicombe, 2001). C-fiber involvement in the cough production has been discussed in previous studies (Mutolo et al., 2009; Canning and Mori, 2011). The cough reflex is subserved by several central structures (e.g., Kubin and Davies, 1995; Gestreau et al., 1997; Bongianni et al., 1998; Pantaleo et al., 2002; Shannon et al., 2004; Bolser et al., 2006; Bonham et al., 2006; Kubin et al., 2006; Jakuš et al., 2008; Poliacek et al., 2011) including the expiratory neurons of the caudal ventral respiratory group (cVRG). These neurons are involved in the production of both the components of the cough motor pattern, but appear to represent merely an expiratory output system for sneezing (Bongianni et al., 2005; Mutolo et al., 2009).

Expiratory neurons of the cVRG have been proved to receive a potent bicuculline-sensitive GABAergic gain modulation that controls their output patterns (McCrimmon et al., 1997; Tonkovic-Capin et al., 2001, 2003). This gain modulation has been suggested to be relevant not only to the control of the breathing pattern, but also to other non-breathing behaviors requiring strong neuronal activation, such as coughing, sneezing, and vomiting. Although the activation of centrally located GABA_B_ receptors, including those within the cVRG and the caudal aspect of the nucleus tractus solitarii (NTS), has been shown to be involved in the control of the cough reflex (Bolser et al., 1994, 1995; Mutolo et al., 2008b, 2010), no information is available, to our knowledge, on the role of brainstem GABA_A_ receptors in this reflex.

The present study was undertaken on pentobarbital sodium-anesthetized, spontaneously breathing rabbits with the main purpose of investigating the role of GABA_A_ receptors within the cVRG in the modulation of coughing induced either by mechanical or chemical stimulation of the tracheobronchial tree. An attempt was also made to investigate the modulatory role of these receptors on the sneeze reflex, a defensive motor act that shares many common features with the cough reflex (Korpáš and Tomori, 1979; Tatar et al., 1988; Wallois and Macron, 1994; Wallois et al., 1995a; Shannon et al., 1997; Pantaleo et al., 2002; Kunibe et al., 2003) and, in particular, the activation of expiratory premotor neurons within the cVRG (Price and Batsel, 1970; Batsel and Lines, 1975, 1978; Korpáš and Tomori, 1979; Jakuš et al., 1985; Shannon et al., 1997).

The results provide evidence that a potent GABA_A_-mediated inhibitory control is exerted at the level of the cVRG not only on baseline respiratory activity, but also on cough and sneeze reflex responses. For these and our previous results on respiratory reflexes, we are in debt to John Widdicombe who included us in a “task force” for the study of cough and encouraged us very much to engage in cough research. He was an outstanding mentor for most of us and left an indelible trace in our life. It is a great pleasure and privilege for us to take part in this special issue dedicated to the memory of Professor Widdicombe.

## Materials and methods

### Animal preparation

Experiments were performed on 12 male New Zealand white rabbits (2.7–3.4 kg) anesthetized with pentobarbital sodium (40 mg kg^−1^ i.v., supplemented by 2–4 mg kg^−1^ every 30 min; Sigma-Aldrich, St. Louis, MO, USA). Atropine (0.15 mg kg^−1^ i.m.) was administered to reduce mucosal secretion in the airways. The adequacy of anesthesia was assessed by the absence of reflex withdrawal of the hindlimb in response to noxious pinching of the hindpaw. Additional criteria were the presence of a stable and regular pattern of phrenic bursts and the absence of fluctuations in arterial blood pressure or phrenic nerve activity, whether spontaneous or in response to somatic nociceptive stimulation. All animal care and experimental procedures were conducted in accordance with the Italian legislation and the official regulations of the European Community Council on the use of laboratory animals (Directive 86/609/EEC). The study was approved by the Animal Care and Use Committee of the University of Florence. All efforts were made to minimize both the number of animals used and their suffering. Experimental procedures and details about the methods employed have previously been described (Bongianni et al., 2005; Mutolo et al., 2007, 2008a,b, 2009, 2010, 2012).

### Recording procedures

Efferent phrenic nerve activity was recorded using bipolar platinum electrodes from the central stump of cut and desheathed C_3_ or C_5_ phrenic roots. The electromyographic (EMG) activity of abdominal muscles was recorded by wire electrodes (Nichrome wires, insulated except for 1 mm at the tips, diameter 0.1 mm) inserted into the external or the internal oblique abdominal muscles. Phrenic and abdominal activities were amplified, full-wave rectified, and “integrated” (low-pass RC filter, time constant 100 ms). Extracellular recording from medullary neurons were performed with tungsten microelectrodes (5–10 MΩ impedance at 1 kHz). The most rostral extent of the area postrema on the midline was defined as the obex and used as a reference point. Neuronal activity was recorded from expiratory neurons of the cVRG (1.6–3.0 mm caudal to the obex, 2.0–2.5 mm lateral to the midline, and 2.0–2.6 mm below the dorsal medullary surface). Arterial blood pressure was recorded by a strain-gauge manometer. End-tidal CO_2_ partial pressure was measured by an infrared CO_2_ analyzer (Datex, CD-102; Normocap, Helsinki, Finland). Integrated phrenic and abdominal activities as well as the signals of the other variables studied were recorded on an eight-channel rectilinearly writing chart recorder (model 8K20; NEC San-ei, Tokyo, Japan). Cardiorespiratory variables were also acquired and analyzed using a personal computer, equipped with an analog-to-digital interface (Digidata 1200, Axon Instruments, Union City, CA, USA) and appropriate software (Axoscope, Axon Instruments).

### Microinjection procedures

**Figure 1:**
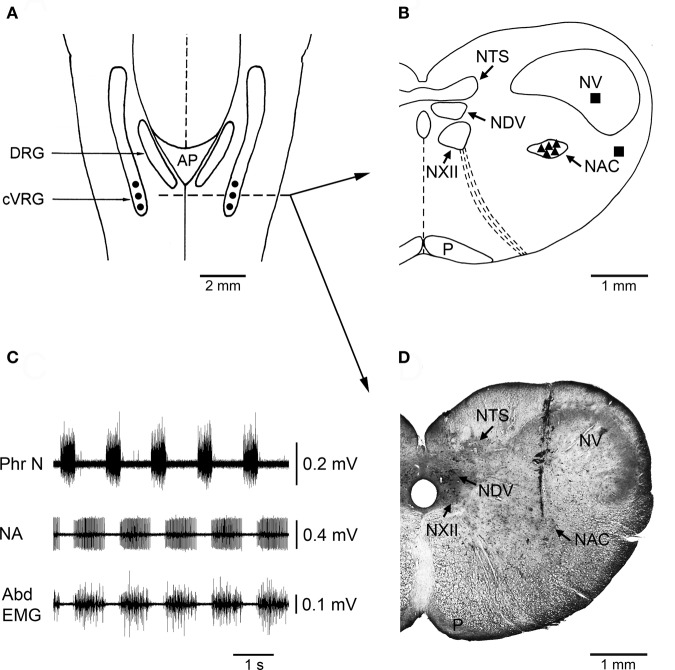
**Localization of injection sites and histological control. (A)** A diagrammatic representation of a dorsal view of the medulla oblongata of the rabbit showing where bilateral microinjections of bicuculline or muscimol were performed into the cVRG (•). AP, area postrema; cVRG, caudal ventral respiratory group; DRG, dorsal respiratory group. **(B)** Diagram of a coronal section of the medulla oblongata at the level indicated in panel A (dashed line) showing the location of representative sites (▲) where the microinjections of 1 mM bicuculline were performed. The diagram also shows the location of some control injection sites (■) where bicuculline caused no appreciable changes in the pattern of breathing and cough responses. NAC, nucleus ambiguus caudalis; NDV, nucleus dorsalis nervi vagi; NTS, nucleus tractus solitarii; NV, nucleus tractus spinalis nervi trigemini; NXII, nucleus nervi hypoglossi; P, tractus pyramidalis. The atlas of Meessen and Olszewski (1949) was used for comparison. **(C)** An example of neuronal expiratory activity recorded in the cVRG region. Phr N, phrenic neurogram; NA, neuronal activity; Abd EMG, abdominal electromyographic activity. **(D)** Photomicrograph of a coronal section of the medulla oblongata at approximately the same level as in B showing the location of a track along which a 1 mM bicuculline microinjection was made into the cVRG. Intense multiunit expiratory activity was recorded at this injection site.

Bilateral microinjections were performed into the cVRG at sites defined by stereotaxic coordinates derived from prior extracellular recordings. The point of entrance of the tungsten microelectrode was visible for a long time after recordings, especially if it was marked by means of a dye (e.g. Cresyl Violet or Pontamine Sky Blue). Therefore, we at first recorded expiratory neuronal activity and marked the electrode entrances. Then, in addition to co-ordinates, we used them as reference landmarks for micropipette placements at the depth where expiratory neurons were recorded. Microinjections (30–50 nl) were performed via a glass micropipette (tip diameter 10–25 μm) by applying pressure using an air-filled syringe connected to the micropipette by polyethylene tubing. The volume of the injectate was measured directly by monitoring the movement of the fluid meniscus in the pipette barrel with a dissecting microscope equipped with a fine reticule. The time taken to inject the solution ranged from 5 to 10 s. In each experiment, at first the rostral limit of the cVRG was determined by recording neuronal activity within the adjacent VRG where a mix of expiratory and inspiratory neurons is encountered (transitional area), approximately from 0.8 to 1.5 mm caudal to the obex. Successively, bilateral microinjections of the selected drugs were made in the cVRG at three different sites, starting from approximately 0.5 mm caudal to the transitional area and continuing along the rostrocaudal extent of the VRG subregion at intervals of 0.5 mm. This procedure was followed to affect as much as possible the entire population of caudal expiratory neurons. Bilateral microinjections at the three selected sites were performed in succession by using a single micropipette. The time taken to perform all the microinjections ranged from 6 to 8 min. The localization of injection sites is diagrammatically illustrated on a dorsal view of the medulla oblongata of the rabbit in Figure 1. The following drugs were used: bicuculline methiodide (a GABA_A_ receptor antagonist; Sigma–Aldrich) and muscimol (a GABA_A_ receptor agonist; Tocris Bioscience, Bristol, UK). Each drug was dissolved in 0.9% NaCl solution. Only one of these drugs was tested in each preparation, unless otherwise stated. Drug concentrations were selected in preliminary trials. They were in the same range as those previously reported to be effective and selective at GABA_A_ receptors (Callera et al., 1999; Bongianni et al., 2010). Control injections of equal volumes of the vehicle solution were also made.

### Stimulation procedures

Cough was induced by means of a 0.5 mm diameter nylon fiber with a smoothed tip inserted through a lateral port of the tracheal cannula until the tip was judged to be near the carina and main bronchi (Tomori and Widdicombe, 1969; Bongianni et al., 2005; Mutolo et al., 2007, 2008b, 2009, 2010, 2012). To attain this condition we proceeded as follows. The nylon fiber was arranged in advance so that it just protruded from the caudal opening of the tracheal cannula, and its length was marked. The total length of the trachea (from the larynx to the carina) was ~6 cm as determined by means of post-mortem measurements performed at the end of some previous experiments. During each experiment the trachea was cannulated for ~3 cm. The nylon fiber was inserted as far as the marked point and subsequently driven toward the carina. Back and forth movements of the fiber (~3 cm) aimed at touching repeatedly (~1 time every s) the carina or nearby airway walls were made over periods of 4–5 s. An interval of ~1 min was scheduled between cough stimulations. As a rule, three stimulation trials were performed in succession before drug administration. These stimulation trials were also executed ~5 min after the completion of all the microinjections and repeated at appropriate intervals (at least 4–5 min). Sneezing was induced by using a 0.3 mm diameter nylon fiber with a smoothed tip inserted into one nostril and pushed two times forward 1.5 cm into the nose. This mechanical stimulation was gentle and short-lasting (~3 s) to avoid as much as possible traumatic effects. Before nasal stimulation, the nylon fiber was positioned into one nostril for an extent (starting point) proved in preliminary trials to be suitable for the generation of consistent reflex responses (Korpáš and Tomori, 1979; Mutolo et al., 2009, 2012). The sneeze reflex (three stimulation trials) was elicited after mechanically-induced cough before and after drug administration. Chemical stimulation of the tracheobronchial tree was performed by means of citric acid inhalation. Stimulation parameters adequate to obtain consistent cough responses were already employed in previous reports (Mutolo et al., 2009, 2012). Citric acid (1 M, Sigma–Aldrich) was freshly dissolved in 0.9% NaCl solution and nebulized (particle diameter 80% from 0.5 to 8 μm; nebulization rate 0.5 ml min^−1^) via an ultrasonic nebulizer (Projet, Artsana, Grandate, CO, Italy). The opening of the tracheal cannula, through which the rabbits were spontaneously breathing, was exposed to a steady stream of the nebulized citric acid solution for ~3 s. This short stimulation period proved to be adequate to avoid as much as possible tachyphylaxis (Mutolo et al., 2009). The interval between chemical challenges was >10 min (usually 15 min) since similar cough reflexes could be reliably obtained at minimal intervals of 7 min (Mutolo et al., 2009, 2012). Chemical stimulation was always applied 2–3 min after mechanically-induced cough and sneeze reflexes and was performed both before and ~10 min after the completion of the injections. Much care was taken to perform all stimulation procedures always at least ~7 min after each supplemental dose of pentobarbital sodium to avoid the possible immediate influences of the injected bolus on both the breathing pattern and reflex responses. All stimulation manoeuvres were executed always by the same experimenter in order to ensure consistency of stimulation intensity between trials. In addition, this experimenter was unaware of the scheduled treatment. All stimulation procedures were repeated at appropriate intervals to follow the time course of the recovery process for a maximum of 3 h.

### Histology

At the end of each experiment, the brain was perfused via a carotid artery with 0.9% NaCl solution and subsequently with 10% formalin solution. After at least a 48 h immersion in 10% formalin solution, the brain was placed in a hypertonic sucrose solution. Frozen 20 μm coronal sections stained with Cresyl Violet were used for the histological control of pipette tracks and injection sites. The atlas of Meessen and Olszewski (1949) was used for comparison. An example of typical placement of the micropipette tip is illustrated in Figure 1 (see also Bongianni et al., 2005; Mutolo et al., 2010).

### Data collection and analysis

Respiratory variables were measured during eupneic breathing and reflex responses (e.g., Mutolo et al., 2012). The inspiratory (T_I_) and expiratory (T_E_) times, as well as the total duration of the respiratory or cough cycle (*T_T_*) were measured on recordings of raw phrenic nerve activity. The respiratory frequency was subsequently calculated (breaths min^−1^). Peak amplitude (arbitrary units) of the phrenic nerve activity and abdominal EMG activity were measured on integrated traces. Normalization of the amplitudes of phrenic and abdominal activities was performed by expressing them as a fraction (or percentage) of the highest achievable amplitude observed in each animal. The highest peak values were consistently observed during coughing. Therefore, all amplitudes have been expressed in relative units (RU; e.g., Bolser et al., 1999; Mutolo et al., 2007, 2008a,b, 2009, 2010, 2012). Breathing pattern variables were measured for an average of five consecutive breaths prior to and following bilateral microinjections of drugs into the cVRG. Furthermore, systolic and diastolic blood pressures were measured at 2 s intervals; mean arterial pressure was calculated as the diastolic pressure plus one-third of the pulse pressure. Average values of cardiorespiratory variables observed under control conditions and at the time when the maximum response occurred (i.e., ~10 min following microinjections; see “Results”) were considered for statistical analyses (Sigma Stat, Jandel Scientific Software, San Rafael, CA). Owing to the small variations in respiratory and cardiovascular variables within each measurement period, average values were taken as single measurements for the purpose of analysis.

The cough motor pattern in response to mechanical or chemical stimulation of the tracheobronchial tree is usually characterized by repeated coughs. Each cough consists of an augmented phrenic burst (preparatory inspiration) immediately followed by a burst of expiratory abdominal activity (Bongianni et al., 2005; Mutolo et al., 2007, 2008b, 2009, 2010, 2012). In agreement with our previous results, repeated coughs usually started during stimulation and continued shortly after stimulus cessation. Respiratory variables of coughs (cough-related variables) included cough-related *T_T_*, *T_I_*, and *T_E_*, peak phrenic amplitude (RU), peak abdominal activity (RU) and cough number, i.e., the number of coughs following each stimulation. Cough-related variables were measured and averaged before and after drug administration (three trials for mechanical stimulation and a single trial for citric acid inhalation). The average values of cough-related variables were taken as single measurements for subsequent statistical analysis. In some cases, the first obvious response following mechanical stimulation of the tracheobronchial tree was a small-amplitude expiratory effort without a preceding preparatory inspiration (Bongianni et al., 2005; Mutolo et al., 2007, 2010). This pattern could fit more appropriately the definition of expiration reflex that is typically evoked by mechanical stimulation of the vocal folds (Korpáš and Tomori, 1979; Widdicombe and Fontana, 2006), but that can be also produced by mechanical stimulation of the tracheobronchial tree (Widdicombe, 1954; Tatar et al., 2008). For further details on this topic see our previous reports (Mutolo et al., 2007, 2008b, 2009, 2010). However, in our study an expiration reflex only occurred as the first motor event in a cough epoch, and its appearance was limited to a few occasions. Therefore, these expiratory responses were not considered for data analysis. Sneezing responses induced by mechanical stimulation consisted of an attack of 3–5 sneezes. Each sneeze consisted of a preparatory augmented inspiration, followed by an intense burst of expiratory activity (Korpáš and Tomori, 1979; Wallois et al., 1995b; Mutolo et al., 2009, 2012). For simplicity, we considered only some sneeze-related variables, i.e., the number of expiratory thrusts or sneeze number, peak phrenic activity and peak abdominal activity. Sneeze-related variables were measured and averaged (three trials) before and after drug microinjections. Also in this case, the average values were taken as single measurements for the purpose of analysis. Average values of cough- and sneeze-related variables observed under control conditions and at the time when the maximum response to microinjections occurred (~10 min) were considered for statistical comparisons. Student's paired *t*-tests were used. All reported values are means ± SE; *P* < 0.05 was taken as significant.

## Results

### Effects of bicuculline microinjections

**Figure 2:**
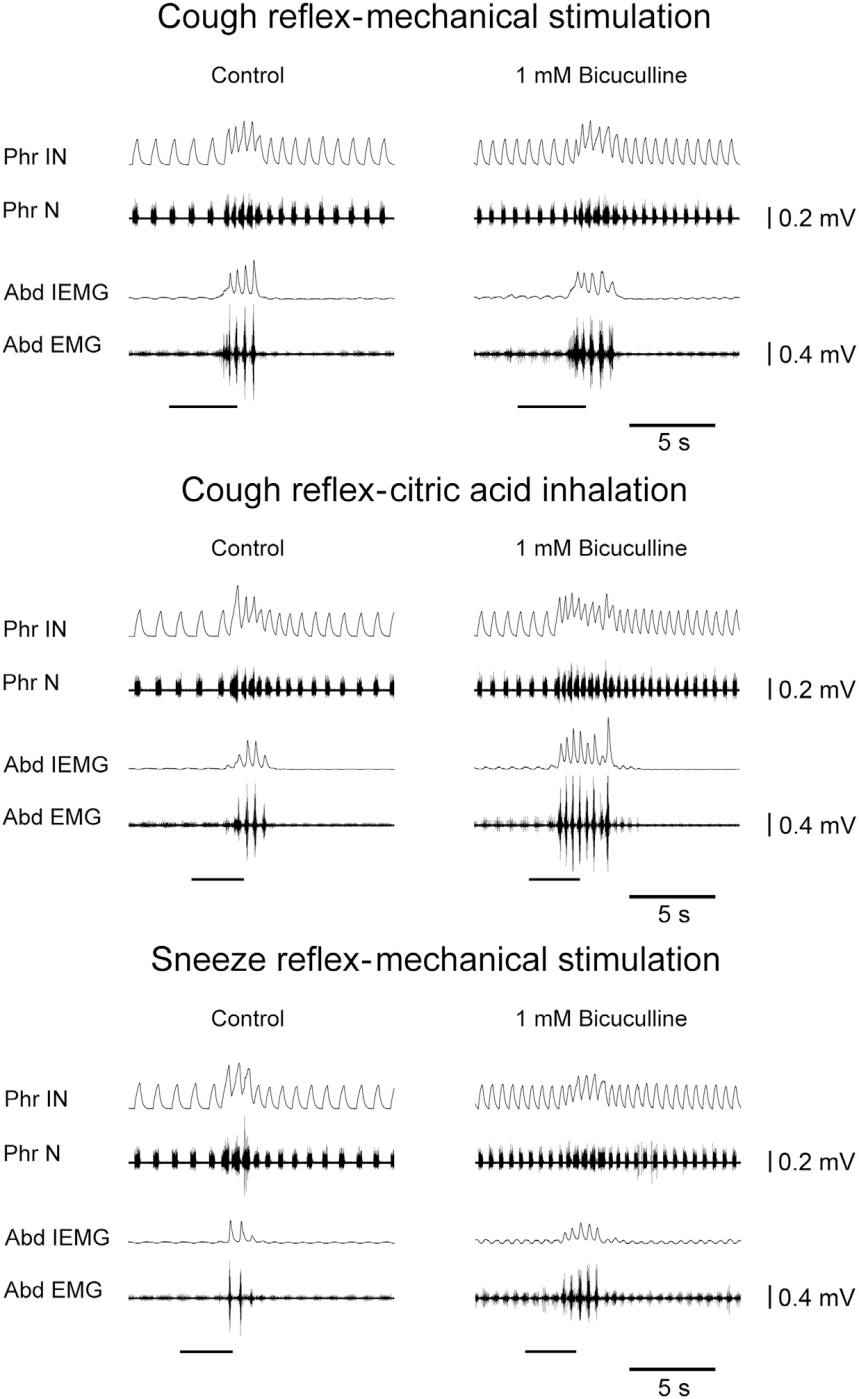
**Potentiating effects on the cough reflex and on the sneeze reflex ~10 min after bilateral microinjections of bicuculline into the cVRG of one anesthetized spontaneously breathing rabbit.** Stimulation periods marked by filled bars. Phr IN, phrenic integrated neurogram; Phr N, phrenic neurogram; Abd IEMG, abdominal integrated electromyographic activity; Abd EMG, abdominal electromyographic activity.

Bilateral microinjections (*n* = 5) of 1 mM bicuculline (30–50 nl; 30–50 pmol) at the selected sites of the cVRG increased peak abdominal activity and respiratory frequency (from 51.2 ± 2.6 to 100.2 ± 9.5 breaths min^−1^; *P* < 0.005) due to decreases in *T_E_* (Table 1). Changes in respiratory activity developed progressively and reached their maximum within ~10 min, while recovery occurred within ~2 h. No significant changes in arterial blood pressure were observed (Table 1). End-tidal CO_2_ partial pressure decreased from 27.07 ± 0.72 to 22.54 ± 0.81 mmHg (*P* < 0.05). Changes in cough-related variables 10 min after bilateral microinjections of 1 mM bicuculline are reported in Table 2. Cough responses caused by mechanical stimulation of the tracheobronchial tree displayed increases in the cough number, while cough responses induced by citric acid inhalation displayed increases both in the cough number and peak abdominal activity associated with decreases in the cough-related *T_T_* due to reductions in both *T_I_* and *T_E_* (Table 2 and Figure 2). Bicuculline microinjections also caused concomitant changes in sneeze-related variables. Increases in the number of the expiratory thrusts and in peak abdominal activity, without significant changes in peak phrenic activity were observed (Table 3 and Figure 2). The recovery of both cough and sneeze responses was obtained within ~2 h.

**Table 1:** **Cardiorespiratory variables during eupneic breathing before and ~10 min after bilateral microinjections of 1 mM Bicuculline, 0.3 mM Muscimol, and vehicle solution into the cVRG**.

	***T_T_, s***	***T_I_, s***	***T_E_, s***	**PPA, RU**	**PAA, RU**	**MAP, mmHg**
**BICUCULLINE (*n* = 5)**						
Control	1.18 ± 0.05	0.39 ± 0.03	0.79 ± 0.08	0.56 ± 0.04	0.08 ± 0.02	98.9 ± 5.1
1 mM	0.62 ± 0.06^**^	0.30 ± 0.03	0.32 ± 0.02^**^	0.44 ± 0.03	0.18 ± 0.04^*^	98.6 ± 6.2
**MUSCIMOL (*n* = 5)**						
Control	1.15 ± 0.05	0.39 ± 0.03	0.75 ± 0.06	0.58 ± 0.04	0.09 ± 0.02	99.3 ± 4.8
0.3 mM	1.58 ± 0.09^**^	0.46 ± 0.02	1.13 ± 0.09^**^	0.56 ± 0.02	0^*^	100.2 ± 6.6
**VEHICLE SOLUTION (*n* = 3)**						
Control	1.17 ± 0.05	0.37 ± 0.02	0.80 ± 0.04	0.55 ± 0.06	0.07 ± 0.02	98.5 ± 4.9
0.9% NaCl	1.18 ± 0.04	0.38 ± 0.02	0.79 ± 0.03	0.58 ± 0.04	0.08 ± 0.01	99.3 ± 4.3

Values are means ± SE; n, number of animals; cVRG, caudal ventral respiratory group; T_T_, cycle duration; T_I_, inspiratory time; T_E_, expiratory time; PPA, peak phrenic activity in relative units (RU); PAA, peak abdominal activity in relative units (RU); MAP, mean arterial blood pressure.

* *P < 0.05*,

** P < 0.005 compared with controls.

**Table 2:** **Changes in cough-related variables recorded ~10 min following bilateral microinjections of 1 mM Bicuculline, 0.3 Muscimol, and vehicle solution into the cVRG**.

	**CN**	***T_T_, s***	***T_I_, s***	***T_E_, s***	**PPA, RU**	**PAA, RU**
**BICUCULLINE (*n* = 5)**						
Mechanical stimulation						
Control	3.6 ± 0.24	0.52 ± 0.03	0.37 ± 0.02	0.15 ± 0.01	0.61 ± 0.04	0.57 ± 0.02
1 mM	4.4 ± 0.24^*^	0.49 ± 0.04	0.33 ± 0.04	0.15 ± 0.02	0.62 ± 0.06	0.59 ± 0.02
Citric acid inhalation						
Control	5.0 ± 0.32	0.52 ± 0.01	0.33 ± 0.01	0.19 ± 0.01	0.63 ± 0.03	0.46 ± 0.02
1 mM	7.2 ± 0.37^**^	0.39 ± 0.01^**^	0.25 ± 0.01^**^	0.14 ± 0.02^*^	0.63 ± 0.02	0.61 ± 0.02^**^
**MUSCIMOL (*n* = 5)**						
Mechanical stimulation						
Control	3.8 ± 0.21	0.54 ± 0.02	0.36 ± 0.02	0.17 ± 0.01	0.64 ± 0.03	0.54 ± 0.02
0.3 mM	–	–	–	–	–	–
*Citric acid inhalation*						
Control	5.2 ± 0.37	0.53 ± 0.02	0.35 ± 0.01	0.18 ± 0.01	0.60 ± 0.04	0.47 ± 0.02
0.3 mM	–	–	–	–	–	–
**VEHICLE SOLUTION (*n* = 3)**						
Mechanical stimulation						
Control	3.7 ± 0.33	0.51 ± 0.05	0.36 ± 0.04	0.15 ± 0.01	0.62 ± 0.04	0.56 ± 0.03
0.9% NaCl	3.6 ± 0.31	0.50 ± 0.05	0.37 ± 0.04	0.14 ± 0.01	0.63 ± 0.08	0.58 ± 0.02
Citric acid inhalation						
Control	5.0 ± 0.58	0.51 ± 0.02	0.34 ± 0.02	0.17 ± 0.01	0.68 ± 0.02	0.46 ± 0.04
0.9% NaCl	5.2 ± 0.44	0.51 ± 0.01	0.35 ± 0.01	0.16 ± 0.01	0.67 ± 0.02	0.45 ± 0.03

Values are means ± SE; n, number of animals; cVRG, caudal ventral respiratory group; CN, cough number; T_T_, cycle duration; T_I_, inspiratory time; T_E_, expiratory time; PPA, peak phrenic activity in relative units (RU); PAA, peak abdominal activity in relative units (RU); –, cough response absent or barely visible (non-measurable).

* *P < 0.05*,

** P < 0.005 compared with control coughs.

**Table 3:** **Changes in some sneeze-related variables recorded ~10 min following bilateral microinjections of 1 mM Bicuculline, 0.3 Muscimol, and vehicle solution into the cVRG**.

	**SN**	**PPA, RU**	**PAA, RU**
**BICUCULLINE (*n* = 5)**			
Control	3.4 ± 0.25	0.72 ± 0.04	0.44 ± 0.05
1 mM	4.8 ± 0.22^*^	0.66 ± 0.03	0.49 ± 0.06^*^
**MUSCIMOL (*n* = 5)**			
Control	3.8 ± 0.32	0.67 ± 0.03	0.46 ± 0.02
0.3 mM	–	0.63 ± 0.02	–
**VEHICLE SOLUTION (*n* = 3)**			
Control	3.5 ± 0.31	0.68 ± 0.04	0.45 ± 0.05
0.9% NaCl	3.4 ± 0.28	0.69 ± 0.04	0.47 ± 0.04

Values are means ± SE; n, number of animals; cVRG, caudal ventral respiratory group; SN, sneeze number; PPA, peak phrenic activity in relative units (RU); PAA, peak abdominal activity in relative units (RU); −, sneeze response absent or barely visible (non-measurable).

* P < 0.05 compared with control sneeze.

### Effects of muscimol microinjections

**Figure 3:**
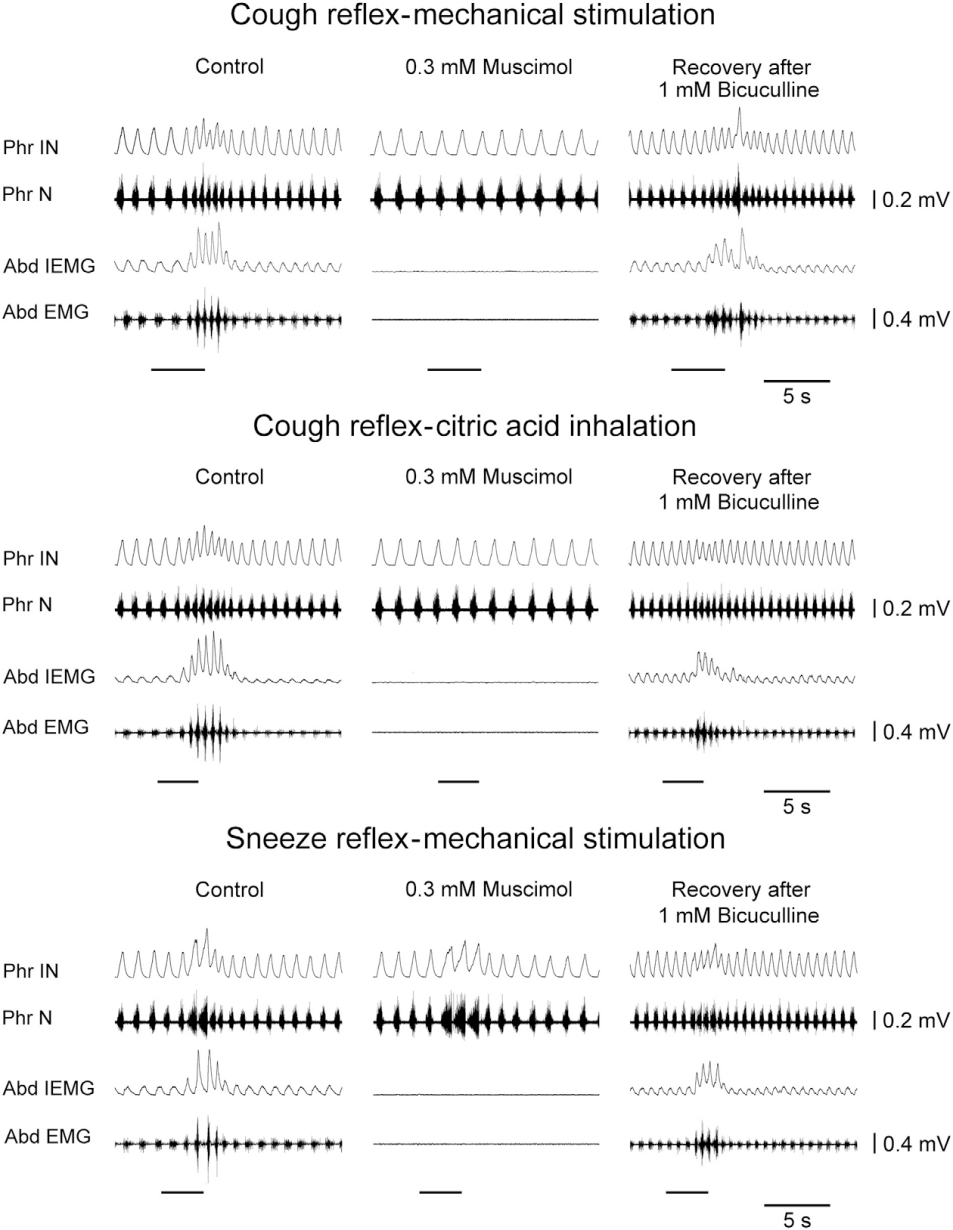
**Depressant effects on the cough reflex and on the sneeze reflex ~20 min following bilateral microinjections of muscimol into the cVRG of one anesthetized spontaneously breathing rabbit.** The recovery of reflex responses ~5 min after 1 mM bicuculline microinjections is reported. Stimulation periods marked by filled bars. Phr IN, phrenic integrated neurogram; Phr N, phrenic neurogram; Abd IEMG, abdominal integrated electromyographic activity; Abd EMG, abdominal electromyographic activity.

Bilateral microinjections (*n* = 5) of 0.3 mM muscimol (30–50 nl; 9–15 pmol) into the cVRG decreased respiratory frequency (from 52.4 ± 2.3 to 38.5 ± 2.7 breaths min^−1^; *P* < 0.005) due to increases in *T_E_* and abolished abdominal activity (Table 1). Changes in respiratory activity developed progressively, reached a maximum within ~10 min and displayed partial recovery after ~3 h. Arterial blood pressure did not change (Table 1). End-tidal CO_2_ partial pressure increased from 26.80 ± 0.77 to 33.22 ± 0.66 mmHg (*P* < 0.05). Cough responses induced by both mechanical and chemical stimulation of the tracheobronchial tree were progressively reduced to barely appreciable single coughs or completely suppressed within ~20 min following muscimol microinjections (Table 2 and Figure 3). Correspondingly, sneeze reflex responses displayed progressive decreases until the expiratory thrusts were hardly visible or abolished, while the preparatory inspiratory bursts persisted without significant changes in amplitude (Table 3 and Figure 3). Cough and sneeze responses recovered, although not completely, after ~3 h (*n* = 2) or within ~5 min following 1 mM bicuculline microinjections at the same cVRG locations (*n* = 3).

### Controls

The localization of the injection sites was confirmed by histological control. Figure 1 illustrates the localization of injection sites on a dorsal view of the medulla oblongata as well as the distribution of injection sites within the cVRG along with an example of typical placement of the micropipette tip. For simplicity, only the distribution of sites where 1 mM bicuculline was injected has been reported. Bilateral control injections (three trials) of equal volumes of the vehicle solution at the selected sites were ineffective (Tables 1 and 2). In two additional animals, bilateral microinjections of bicuculline (*n* = 2) or muscimol (*n* = 3) at medullary locations sufficiently far (> 0.8 mm) from the responsive sites failed to induce the characteristic effects reported above (see Mutolo et al., 2010). The control microinjections of bicuculline were performed into the nucleus tractus spinalis nervi trigemini and the reticular formation lateral to the cVRG, while those of muscimol were performed into the nucleus tractus spinalis nervi trigemini, the reticular formation lateral to the cVRG and caudal to the cVRG, i.e., caudal to the nucleus ambiguus caudalis (see Figure 1 and for comparisons the atlas of Meessen and Olszewski, 1949).

## Discussion

This study is the first to provide evidence that GABA_A_ receptors within the cVRG mediate a potent inhibitory modulation on the pattern of breathing and, more interestingly, on both cough and sneeze reflex responses. This GABAergic inhibition is already exerted under basal conditions. The specificity of GABA_A_ receptor-mediated modulation is substantiated by the responses to microinjections of the specific GABA_A_ receptor agonist muscimol. These responses were in the opposite direction to those produced by bicuculline as would be expected from antagonism based on the same mechanism. In addition, muscimol-induced effects were reverted by bicuculline microinjections at the same locations.

We have already provided details about the microinjection techniques used, as well as a discussion on their reliability and on the spread of the injectate (e.g., Bongianni et al., 2005; Mutolo et al., 2007, 2008b, 2009, 2010). Injection sites were selected by using stereotaxic coordinates and, especially, extracellular recordings from expiratory neurons of the cVRG (Jiang and Shen, 1991; Bongianni et al., 2005; Mutolo et al., 2010; reviewed in Von Euler, 1986). Their localization was confirmed by the histological control. Our previous observations on the spread of the injectate ≤50 nl (Mutolo et al., 2002, 2005) are in agreement with theoretical calculations by Nicholson (1985) suggesting that a volume of 50 nl should spread <385 μm in any direction from the injection site. Accordingly, drug microinjections into regions sufficiently away from the responsive sites did not cause the above reported effects. The specificity of the effects obtained is also supported by the absence of changes in the ongoing pattern of breathing and reflex responses following control bilateral microinjections of the vehicle solution.

Bicuculline- and muscimol-induced effects on the intensity of the expiratory motor output are clearly related to disinhibition and inhibition of cVRG expiratory premotor neurons, respectively. These neurons are known to be the target of GABA_A_ receptor-mediated inhibition (McCrimmon et al., 1997; Tonkovic-Capin et al., 2001, 2003). The possible sources of inhibitory inputs to these neurons, although not completely known, have been summarized by Iscoe (1998). They comprise early inspiratory, postinspiratory and late inspiratory neurons of the VRG as well as late inspiratory neurons of the ventrolateral NTS. In addition, Bötzinger complex expiratory neurons send inhibitory projections to cVRG expiratory neurons (see also Bongianni et al., 1997). The interpretation of changes in respiratory frequency induced by the two drugs is far more complex. It implies that neurons located in the cVRG project to more rostral ponto-medullary regions widely considered to be responsible for respiratory rhythm generation and pattern formation (Von Euler, 1986; Feldman and Del Negro, 2006; Abdala et al., 2009). These regions include the rostral VRG, the parabrachialis medialis/Kölliker Fuse nuclei and the NTS (Smith et al., 1989; Gerrits and Holstege, 1996; Iscoe, 1998; Zheng et al., 1998). This interpretation is in agreement with previous findings obtained with chemical activation of cVRG neurons in rats (Bonham and Jeske, 1989; Chitravanshi and Sapru, 1999), cats (Bongianni et al., 1994; Poliacek et al., 2007) and rabbits (Bongianni et al., 2005). It is not known which cVRG neurons send these projections. Although changes in the intensity of expiratory activity suggest an involvement of cVRG expiratory bulbospinal neurons, other types of cVRG neurons either quiescent or with different respiratory or non-respiratory discharge patterns (Arita et al., 1987; Jiang and Shen, 1991; Iscoe, 1998) may have a role. How and where the influences of these different types of neurons are exerted is, at present, just matter of speculation. Previous studies have shown that chemical stimulation of cVRG neurons induced by excitatory amino acids in different animal species (Bonham and Jeske, 1989; Bongianni et al., 1994, 2005; Chitravanshi and Sapru, 1999; Poliacek et al., 2007) causes depressant effects on inspiratory activity. Present results are at variance with these previous findings since excitation on cVRG neurons due to bicuculline-induced disinhibition causes strong increases in respiratory frequency, whilst muscimol-induced inhibition of the same population of neurons provokes decreases in respiratory frequency. The reason of this discrepancy is not clear, but in the interpretation of these effects it should be taken into consideration that chemical stimulation affects all types of neurons, while GABA_A_ receptor agonists and antagonists affect only neurons receiving GABAergic inhibition, possibly a special group of neurons involved in reflex changes in respiratory frequency (e.g., Sant'Ambrogio and Widdicombe, 2001). In particular, the finding that bicuculline-induced changes in respiratory frequency were mainly due to decreases in *T_E_* could suggest that ascending projections from cVRG neurons impinge on second- or third-order neurons of the afferent pathway from RARs and C-fiber receptors, whose activation has been shown to induce decreases in *T_E_* (Lee and Pisarri, 2001; Sant'Ambrogio and Widdicombe, 2001). Interestingly, an inhibitory modulation of the cough reflex could also be exerted at the level of NTS, where second-order neurons of the afferent pathway from RARs receive a GABAergic and possibly glycinergic inhibitory input from pump cells (Ezure et al., 1999; Ezure and Tanaka, 2000, 2004). In this context, it should also be recalled that bicuculline-induced effects on respiratory frequency could be potentiated by the peripheral vagal feedback from pulmonary stretch receptors. In fact, in the presence of excitatory effects on inspiratory activity (increases in drive and therefore in the slope of inspiratory activity) the vagal feedback implies a low inspiratory off-switch threshold, decreases in *T_I_* and consequent increases in frequency (Von Euler, 1986). However, the role of pulmonary stretch receptor afferents is questionable given the absence of significant changes in peak phrenic activity and T_I_, and therefore in the slope of inspiratory activity.

Given the potent GABA_A_ receptor-mediated modulation of basal expiratory activity and of expiratory thrusts during cough and sneeze responses, it seems conceivable that the observed bicuculline- and muscimol-mediated effects rely, to a great extent, on changes in the excitability of bulbospinal expiratory neurons. However, since the inspiratory component of the cough reflex is also affected by muscimol, we have to admit, in agreement with our previous findings (Bongianni et al., 2005; Mutolo et al., 2009; see also Mutolo et al., 2010), that this cannot be simply explained by the activation of GABA_A_ receptors located on cVRG expiratory neurons. Bilateral lesions of descending bulbospinal expiratory pathways within the ventral columns of the cervical spinal cord impair spontaneous rhythmic abdominal activity and interfere only with the expiratory components of the cough response (Newsom Davis and Plum, 1972). Accordingly, the inhibition of other types of cVRG neurones (Arita et al., 1987; Jiang and Shen, 1991; Iscoe, 1998), possibly receiving cough-related inputs and projecting to brainstem respiration-related regions (Smith et al., 1989; Gerrits and Holstege, 1996; Iscoe, 1998; Zheng et al., 1998), may have contributed to the observed effects.

The results show that the most significant change following bicuculline microinjections is the increase in the cough number. Increases in the cough number could appear to be related to increases in respiratory frequency. However, both phenomena are dependent upon bicuculline microinjections and even if a correlation between them could be proved, it does not imply any inference on a possible relationship of cause and effect. In addition, sampled data are relatively few for an appropriate interpretation. In a previous study on the cough reflex responses during pulmonary C-fiber receptor activation (Mutolo et al., 2008a), we have provided evidence supporting the hypothesis that the neural mechanisms generating cough are dependent upon the characteristics of the ongoing respiratory activity. In particular, the timing of the cough motor pattern but not the intensity of peak abdominal activity appears to be, to some extent, related to the timing of baseline respiratory activity, thus implying that the reconfigured respiratory pattern generator during coughing (e.g., Bongianni et al., 1998; Pantaleo et al., 2002; Bolser et al., 2003; Shannon et al., 2004) retains memory of the preceding respiratory pattern. However, in the present study bicuculline-induced changes in cough-related timing variables were seen only during citric acid-induced cough and do not appear to parallel changes in the ongoing respiratory activity. In fact, at variance with baseline respiratory activity, changes in timing concerned not only T_E_, but also T_I_. Furthermore, changes in cough-related variables following muscimol microinjections were dramatically different from those observed in the baseline pattern of breathing. It should also be recalled that the cough number has been suggested to depend more on the activation of a central gate mechanism than on the baseline pattern of breathing (Bolser et al., 1999; Bolser and Davenport, 2002; Bolser et al., 2006). Interestingly, it has been reported that exercise-induced or voluntary hyperpnea exerted inhibitory influences on the cough reflex (Lavorini et al., 2010). In other words, cough will be down-regulated at the expenses of hyperpnea. In conclusion, we are confident that the observed effects on the cough reflex depend, at least to a great extent, on bicuculline-induced disinhibition of cVRG neurons.

In this context, it should also be mentioned that the intensity of the cough reflex is influenced by chemical drive (reviewed in Hanacek et al., 2006; Widdicombe and Singh, 2006). In particular, there is a general consensus that in anesthetized animals and humans acute hypercapnia, hypoxia and asphyxia stimulate breathing, but down-regulate the cough and other defensive reflexes. Furthermore, hypercapnia and hypocapnia have been reported to have little effects on cough in anesthetized humans (Nishino et al., 1989). On the other hand, the role of hypocapnia alone on cough regulation has not been studied (Hanacek et al., 2006). It is apparent that the intensity of cough cannot be directly related to the strength of breathing due to chemical stimuli (Widdicombe and Singh, 2006). Bicuculline-induced increases in frequency lead to decreases in end-tidal CO_2_ partial pressure and therefore in chemical drive. The reverse occur following muscimol microinjections, however, changes in CO_2_ are relatively small and lower than those required to conceivably affect the cough reflex (Hanacek et al., 2006).

The reasons of the observed differences between citric acid- and mechanically-induced cough following bicuculline microinjections could be related to the increased ventilation and, therefore, to the higher number of inhaled nebulized particles. However, we believe that it is not the case. In fact, in preliminary trials performed to obtain adequate parameters for chemical stimulation in the rabbit (Mutolo et al., 2009), we found that cough responses were rather stereotyped and did not show obvious increases by increasing the stimulation period. Indeed, after the first few seconds coughing ceased in spite of persisting chemical stimulation. Thus, 3 s stimulation periods were chosen since they were short but sufficient for the full expression of the citric acid-induced cough reflex and, at the same time, they allowed us to reduce as much as possible tachyphylaxis. Taking into consideration that chemical stimulation conceivably affects a higher number of cough receptors as compared with punctate mechanical stimulation, we propose that it can lead to a larger recruitment of bulbospinal expiratory neurons affected by bicuculline-induced disinhibition. In the present experiments, peak abdominal activity increased only during citric acid-induced cough following bicuculline microinjections. This could appear to be related to the lower abdominal muscle activation under control conditions as compared with that observed during cough induced by mechanical stimulation. However, the level of abdominal activation under control conditions is similar to that we observed in previous studies (Mutolo et al., 2009, 2012) during both mechanically- and chemically-induced cough. In addition, there is no evidence that pentobarbital sodium depresses expiratory motor discharge during cough. The level of excitatory motor drive to expiratory motoneurons during cough is far greater than that present during eupnea and probably overwhelms any depressant effect of the anesthetic (see Bolser et al., 1999 also for further references). We do not know the reason for the lower abdominal muscle activation during control citric acid-induced cough, however, we cannot completely exclude that the observed effects are related, at least in part, to the influences of the anesthesia. At variance with cough, only the expiratory bursts of sneezing were suppressed by muscimol. As already discussed (Mutolo et al., 2009), most probably second-order neurons located within different subnuclei of the spinal trigeminal sensory complex provide the appropriate inputs to the different components of the respiratory network responsible for the generation of the complete sneeze motor pattern (Wallois et al., 1995a; Shannon et al., 1997; Dutschmann et al., 1998). Present results also confirm that both the inspiratory and expiratory components of the cough reflex are, to a large extent, organized at the level of the cVRG (Bongianni et al., 2005; Mutolo et al., 2009).

Recently, the presence of a cough-suppressant mechanism has been revealed within the cVRG (Poliacek et al., 2007, 2010). Our results could suggest that not only GABA_B_ (Bolser et al., 1994, 1995; Poliacek et al., 2007; Mutolo et al., 2010), but also GABA_A_ receptors may play a role in this suppressant mechanism. Our study does not allow us to determine whether the investigated GABA mechanism is acting pre- or post-synaptically. Previous studies have suggested that both pre- and post-synaptic GABA_A_ receptors mediate the inhibitory modulation of cVRG expiratory neurons (McCrimmon et al., 1997; Tonkovic-Capin et al., 2003). The results of the present study may provide hints for further investigations on the modulatory effects on the cough reflex exerted by compounds (e.g., benzodiazepines and barbiturates) acting on GABA_A_ receptors at different brainstem sites (e.g., Chou and Wang, 1975). Interestingly, a pioneering study on the effects of barbiturates on the cough reflex was performed by May and Widdicombe (1954). Investigations on this matter may provide fruitful strategies for the development of novel antitussive therapies.

### Conflict of interest statement

The authors declare that the research was conducted in the absence of any commercial or financial relationships that could be construed as a potential conflict of interest.
